# Diagnoses of children living with HIV before and during the COVID-19 pandemic

**DOI:** 10.4102/sajid.v39i1.652

**Published:** 2024-10-29

**Authors:** Asandiswa L. Shange, Lisa J. Frigati, Moleen Zunza

**Affiliations:** 1 Department of Paediatrics and Child Health, Faculty of Medicine and Health Sciences, Stellenbosch University, Cape Town, South Africa; 2 Division of Epidemiology and Biostatistics, Faculty of Medicine and Health Sciences, Stellenbosch University, Cape Town, South Africa

**Keywords:** paediatrics, HIV, COVID-19, advanced HIV disease, hospitalised

## Abstract

**Background:**

There is limited data on diagnoses during hospital stay among children living with HIV(CLHIV) in the antiretroviral and coronavirus disease 2019 (COVID-19) era.

**Objectives:**

The aim of this study was to describe hospital diagnoses and clinical characteristics of CLHIV before and during the COVID-19 pandemic.

**Method:**

A retrospective descriptive cross-sectional study was performed. Clinical and laboratory data were retrieved by reviewing folders and discharge summaries from January 2019 to December 2021. Period A (pre-COVID-19) was defined as the period from January 2019 to March 2020. Period B (During COVID-19) was defined as being from April 2020 to December 2021.

**Results:**

Ninety-six children contributed 215 diagnoses over the study period. The five most common diagnoses were unspecified HIV disease (47/215, 21.9%), tuberculosis (TB) (42/215, 19.5%), pneumonia (13/215, 6.0%), encephalopathy (11/215, 5.1%) and malnutrition (11/215, 5.1%). Median CD4 count was 377 cells/mm (IQR 126, 726) and 8.0% of the children were virally suppressed. Ninety-five per cent of the children had WHO Stage 3 and 4 (95%) disease and 12.5% of children required ICU admission. No child was diagnosed with COVID-19 despite universal screening. Moreover, 81.7% of the children had a social worker referral documented.

**Conclusion:**

Advanced HIV disease (AHD) remains prevalent with TB being the most common diagnosis. There were no cases of COVID-19 recorded in CLHIV.

**Contribution:**

The findings provide a description of the diagnoses of CLHIV in the South African setting prior to and during the COVID-19 pandemic. It highlights the need for more specific documentation of diagnoses to inform better prevention of AHD in children.

## Introduction

Children living with human immunodeficiency virus (HIV) children living with HIV are hospitalised for AIDS and non-AIDS-related diseases. After the introduction of antiretroviral therapy (ART), AIDS-related morbidity has decreased.^1^

In March 2020, the World Health Organization (WHO) declared the surge in coronavirus disease 2019 (COVID-19) cases a pandemic.^2^ Studies have suggested that during the lockdown period, there was a disturbance of child health care services.^3,4,5^ Three studies that were conducted stated that during the lockdown period, there was a decrease in hospitalisations, clinic visits and immunisations of children.^3,4,5^ The most important reason for the decrease in health seeking behaviour was fear of contracting COVID-19.^3,4^ One study postulated government restrictions and travel expenses during an economically challenging time to be additional reasons.^4^ A study conducted in Durban, South Africa, found that there was no significant difference in the HIV viral loads of children prior to and after this period.^6^ There is also limited data on whether children with HIV are at risk of COVID-19 disease.^7^

Few studies describe the diagnoses during hospital stay of CLHIV. Among studies that have been conducted, bacterial infections, AIDS-related conditions and malnutrition have been the most common documented causes of hospitalisation.^1,8^ A systematic review of studies performed worldwide found bacterial infections to be the most common cause of hospitalisation in CLHIV, AIDS-related conditions as the second and malnutrition to be the fourth most common cause.^1^ Children living with HIV that do not adhere to antiretroviral therapy are at increased risk of acquiring infections as well as developing severe acute malnutrition (SAM).^9^ South Africa is a high TB burden country with an estimated TB incidence rate of 737/100 000 population in 2018.^10^

### Objectives

The aim of this study was to describe the diagnoses during hospital stay of CLHIV admitted to Tygerberg Hospital before and during the COVID-19 pandemic.

## Research methods and design

We conducted a retrospective descriptive cross-sectional study of CLHIV admitted to Tygerberg Hospital. Tygerberg is a tertiary hospital in the Western Cape, South Africa. It is the largest hospital in the province and the second largest in South Africa with a 1899 bed capacity. Three hundred and eight of these beds are allocated to the Tygerberg Children’s Hospital (TCH). Children from the Western Cape, as well as some areas of the Eastern Cape and Northern Cape, requiring specialist care are treated in the hospital. On average, 16 000 children are admitted and 100 000 are treated on an outpatient basis per year. This study was conducted in wards G7 (general paediatrics), G9 (paediatric pulmonology, neurology and endocrinology), G10 (infectious diseases and gastroenterology) and the paediatric intensive care unit (PICU) of TCH. Children presenting to the emergency ward, those requiring admission from the Infectious Diseases Outpatient Clinic and those that are referred from other hospitals requiring specialist infectious disease care are admitted to these wards. The only paediatric wards that were not included were oncology, neonatology and the surgical paediatric wards.

The study period was from January 2019 through to December 2021, and this was further divided into the pre-COVID-19 period (defined as 01 January 2019 – 31 March 2020) and the COVID-19 period (defined as 01 April 2020 – 31 December 2021). Period B was 6 months longer than period A as there were very few hospital admissions during the period of April 2020 to September 2020 when the harshest lockdown measures were implemented. There were no children admitted with HIV in February and March 2020.

The inclusion criteria were any child less than 13 years old with confirmed HIV according to South African National Guidelines.^11^ Exclusion criteria were HIV-negative children.

We included age, sex, HIV viral load, CD4 count, duration of Tygerberg hospital admission, municipality area of residence, admission outcome (discharge, transfer or death), International Classification of Diseases (ICD) code at time of discharge, social worker referral, ART regimen, admission weight and length or height as well as the level of care required by the patient (ward or intensive care). We used weight and height/length measurements to determine WFA (weight for age), LFA (length for age) and WFL (weight for length) WHO Z-scores. The WHO clinical staging classification for HIV/AIDS was used to clinically stage children.^12^

It was unclear how treating clinicians chose the ‘primary’ diagnosis on the discharge summaries, so we did not classify primary versus secondary diagnosis. All the diagnoses documented on the discharge summaries were included to determine what were the most prevalent diagnoses. We grouped together ICD codes that described any clinical presentation of malnutrition, tuberculosis (TB) and pneumonia, respectively.

From April 2020 through May 2020, all children that presented with cough, sore throat, shortness of breath or fever > 38.0°C were tested for Severe acute respiratory syndrome coronavirus 2 (SARS COV-2), and from June 2020 through December 2021, universal testing of any child transferred or admitted from casualty to any of the abovementioned wards was tested for SARS COV-2.^13^

### Data collection and storage

Data for this study were obtained from the Tygerberg Hospital electronic registers (ECM). The ECM is a database containing digital copies of patient clinical records. The data were manually extracted and recorded onto a data collection sheet by the primary investigator. The data were stored electronically on the Stellenbosch University OneDrive.

### Sample size

A sample size of 96 patients was used in this study. As this was a retrospective descriptive study of a defined period, there was no predefined sample size.

### Data analysis

We summarised categorical variables using count and percentage. Continuous variables were summarised using mean (standard deviation) or median (interquartile range) depending on the distribution. We tested association between categorical variables using chi-squared test or Fishers exact test. Comparison of continuous variables between two groups was performed using *t*-test or Mann-Whitney U test. Statistical significance was set at *p*-value < 0.05.

### Ethical considerations

Ethical approval was obtained from the Stellenbosch University Health Research Ethics Committee (HREC) with study approval number U22/05/182. Waiver of consent for this retrospective study was granted by the HREC. The data in this study were aggregated and anonymised. Individual clinical conditions were reported on, with no way of linking them back to the patients.

## Results

Of the 96 children included in this study, 42 (43.8%) were female. The median age was 2.1 years (interquartile range [IQR]: 0.8, 6.3). The median CD4 count was 377 cells/mm^3^ (IQR 126, 726), and 8.0% of the children were virally suppressed (< 50 copies/mL). The majority of children (84.0%) were classified as WHO Stage 4 (Table 1).

**TABLE 1:** Demographics, HIV-related characteristics and post-admission outcomes of children living with HIV admitted to Tygerberg Hospital.

Variable	Total (*N* = 96)				Period A (*n* = 41)				Period B (*n* = 55)			
	*n*	%	Median	IQR	*n*	%	Median	IQR	*n*	%	Median	IQR
Sex (female)	42	43.8	-	-	22	53.7	-	-	20	36.4	-	-
Age (years)	96	-	2.1	0.8 to 6.3	-	-	2.6	1.1 to 6.4	-	-	1.7	0.5 to 5.5
Mother alive	81	87.1	-	-	36	90.0	-	-	45	84.9	-	-
Length of hospital stay (days)	-	-	11	5 to 21	-	-	8	-	-	-	11	-
Weight for age (WFA)	-	-	-	-	-	-	–1.7	–2.9 to –0.7	-	-	−2.9	–3.8 to –2.0
Length or height for age	-	-	-	-	-	-	–2.7	–3.9 to –0.5	-	-	−1.9	–2.8 to –0.5
Weight for length or height	-	-	-	-	-	-	–1.3	–2.9 to –0.1	-	-	−2.2	–3.5 to –0.7
**HIV-related characteristics**												
Newly diagnosed on admission	28	29.2	-	-	13	31.7	-	-	15	27.3	-	-
CD4 count (cell/mm^3^)	-	-	377	126 to 726	-	-	474	210 to 820	-	-	324	62 to 645
Viral load (log)	-	-	5.6	3.8 to 6.3	-	-	5.3	4.0 to 6.0	-	-	5.9	3.8 to 6.5
Viral load < 50 copies/mL	6	8.0	-	-	3	2.6	-	-	3	3.4	-	-
WHO stage												
I	3	3.2	-	-	3	7.5	-	-	0	0.0	-	-
II	1	1.1	-	-	1	2.5	-	-	0	0.0	-	-
III	11	11.7	-	-	5	12.5	-	-	6	11.1	-	-
IV	79	84.0	-	-	31	77.5	-	-	48	88.9	-	-
**Outcome post-admission**												
Discharged home	77	80.2	-	-	37	90.2	-	-	40	72.7	-	-
Transferred to different hospital	16	16.7	-	-	3	7.3	-	-	13	23.6	-	-
Died	3	3.1	-	-	1	2.4	-	-	2	3.6	-	-

WFA, weight for age; IQR, interquartile range; WHO, World Health Organization.

There were 28 children newly diagnosed with HIV on admission (29.2%) and 71.4% of these newly diagnosed children were classified as WHO stage 4. The median CD4 count in the newly diagnosed children was 245 cells/mm^3^ (IQR 49, 462), and the median viral load was log 6.34 (IQR 5.9, 6.7). For those already initiated on ART, the median CD4 count was 532 (IQR 154, 927), and the median viral load was log 4.8 (IQR 2.6, 6.1). Only three children were on Dolutegravir (DTG), and these were >20 kg and 11 years old. Of the three children on DTG, two were suppressed and one was newly diagnosed.

It was found that 3.1% of the children included in this study died (two died in PICU, one in the ward). The causes of death were tuberculous meningitis (TBM), pneumocystis pneumonia (PJP) and a ‘bacterial infection’. The remainder were discharged (80.2%) or transferred to a different facility (16.7%). Eighty-one per cent of children had documentation of being referred to the social worker.

Table 2 summarises the most common diagnoses during hospital stay. There were 215 diagnoses in total. Besides 1 child that had no diagnosis listed, each child had at least one primary diagnosis documented. Only 62 (64.6%) of the 96 children had a second diagnosis, with 33 having a third, and 15 having a fourth, eight having a fifth, two having seven diagnoses and one child had 10 diagnoses listed. The five most common diagnoses were unspecified HIV disease (47/215, 21.8%), TB (42/215, 19.5%), pneumonia (13/215, 6.0%) and encephalopathy (11/215, 5.1%) and malnutrition (11/215, 5.1%). The proportion of diagnoses in Period A and Period B is summarised in Table 2. No child was diagnosed with COVID-19 disease in Period B despite universal screening.

**TABLE 2:** Diagnoses of children living with HIV admitted to Tygerberg Hospital.

Most common diagnoses of children living with HIV	Total % (*N* = 215)	Period A (*n* = 90)	Period B (*n* = 125)
Unspecified HIV disease	47	28	19
HIV resulting in CMV	6	3	3
HIV resulting in candidiasis	5	0	5
Gastroenteritis/colitis of infectious origin	4	2	2
HIV resulting in PJP	6	3	3
Tuberculosis (TB)†	42	15	27
Encephalopathy	11	5	6
Bacterial infections	6	2	4
Malnutrition‡	11	9	2
Pneumonia§	13	5	6

CMV, cytomegalovirus; PJP, pneumocystis pneumonia; TBM, tuberculous meningitis; TB, tuberculous; PEM, protein energy malnutrition; RSV, respiratory syncytial virus.

† , TB: 22: HIV resulting in TB, nine confirmed TB, three unconfirmed, six TBM, two TB of other organs;

‡ , Malnutrition: three cases wasting syndrome, four severe protein energy malnutrition (PEM), two unspecified PEM, one moderate PEM;

§ , Pneumonia: one adenoviral pneumonia, one RSV pneumonia, three other viral pneumonia, one bacterial pneumonia, three bronchopneumonia unspecified, one pneumonia unspecified and three unspecified lower respiratory tract infections.

## Discussion

Ninety-six CLHIV were admitted from January 2019 to December 2021. The most common diagnosis was TB. This finding was expected as TB is a known cause of morbidity among adults and CLHIV.^14,15^ One study found that CLHIV were 24.2 times more likely to have TB in comparison to uninfected infants.^16^

Infections (‘multiple infections’, ‘bacterial infections’ and ‘infectious or parasitic disease’) and AIDS-related infections (Cytomegalovirus, Pneumocystis jirovecii pneumonia and Candidiasis) collectively were found to be common diagnoses during hospital stay, making up 18.6% of overall diagnoses.More data in children with the diagnosis of ‘multiple infections’ is needed to ascertain if these were bacterial or viral. In addition, there was limited laboratory confirmation of infections. There was one Klebsiella pneumoniae bacteraemia documented and one child had adenovirus and another respiratory syncytial virus pneumonia. Of note, no children were diagnosed with COVID-19 disease during Period B despite the hospital being a referral centre for children with COVID-19 disease and 70% of the children not having a supressed HIV viral load.

Despite widespread awareness and knowledge about HIV, advanced HIV remains prevalent, as seen by statistics showing 12.5% of children requiring PICU care and 95% of the cohort with WHO Stage 3 or 4 disease.

The findings of this study are similar to that found in the literature. A systematic review exploring the causes of hospitalisation among people living with HIV found AIDS-related illnesses and bacterial infections to be the leading causes of hospitalisation among the paediatric population.^1^ A cohort study reviewing admissions of adolescents with HIV found TB to be the most common infectious cause of admission.^17^ A case series describing the hospitalisation of CLHIV in Malawi found malnutrition and pneumonia to be the first and second most common causes of hospitalisation.^8^

Tuberculosis remains the most common diagnosis during hospital stay among CLHIV. As these children are known to be high risk for acquiring TB, we recommend screening and preventative strategies.^15,18^ Symptom screening and tuberculin skin test (TST) may be used as screening modalities.^18^ Preventative strategies include the use of the Bacille Calmette-Guerin (BCG) vaccine, TB preventative treatment and ensuring more children have suppressed HIV viral loads through optimisation of antiretroviral treatment.^15^

A high proportion of children were referred to the social worker (81.7%). We recommend greater emphasis and availability of social services to evaluate social circumstances that may be impeding optimal treatment.

### Limitations

As this was a retrospective study, there are some missing data. There are many non-specific diagnoses, and this highlights the need for better documentation of exact diagnoses to enable preventative strategies. We initially wanted to compare diagnoses between period A and B; but because of differing length of the periods, we could not do this.

The findings may be specific to children admitted to the referral hospital and not generalisable to other settings with a lower TB incidence or other prevalent opportunistic infections.

## Conclusion

Advanced HIV disease remains a significant problem in CLHIV. Tuberculosis remains the most common cause of hospital admission among CLHIV. Most children admitted to the hospital during the study period were already diagnosed with HIV prior to admission. No CLHIV was diagnosed with COVID-19 disease over this period. Better interventions are needed to prevent opportunistic infections and ensure that children already diagnosed with HIV can adhere to treatment.

## References

[1] Ford N, Shubber Z, Meintjes G, et al. Causes of hospital admission among people living with HIV worldwide: A systematic review and meta-analysis. Lancet HIV. 2015;2(10):e438–e444. 10.1016/S2352-3018(15)00137-X 26423651

[2] Cucinotta D, Vanelli M. WHO declares COVID-19 a pandemic. Acta Biomed. 2020;91(1):157–160. 10.23750/abm.v91i1.9397 32191675 PMC7569573

[3] Jensen C, McKerrow NH. Child health services during a COVID-19 outbreak in KwaZulu-Natal Province, South Africa. S Afr Med J. 2020;0(0):13185. 10.7196/SAMJ.2021.v111i2.15243 33334393

[4] Siedner MJ, Kraemer JD, Meyer MJ, et al. Access to primary healthcare during lockdown measures for COVID-19 in rural South Africa: An interrupted time series analysis. BMJ Open. 2020;10(10):e043763. 10.1136/bmjopen-2020-043763. Erratum in: BMJ Open. 2020;10(11):1. 10.1136/bmjopen-2020-043763 PMC7536636 33020109

[5] McIntosh A, Bachmann M, Siedner MJ, Gareta D, Seeley J, Herbst K. Effect of COVID-19 lockdown on hospital admissions and mortality in rural KwaZulu-Natal, South Africa: Interrupted time series analysis. BMJ Open. 2021;11(3):e047961. 10.1136/bmjopen-2020-047961 PMC7977076 33737445

[6] Mathamo A, Naidoo KL, Dorward J, Archary T, Bottomley C, Archary M. COVID-19 and HIV viral load suppression in children and adolescents in Durban, South Africa. S Afr J HIV Med. 2022;23(1):1424. 10.4102/sajhivmed.v23i1.1424 PMC9772656 36575700

[7] Berzosa Sánchez A, Epalza C, Navarro ML, et al. SARS-CoV-2 Infection in children and adolescents living with HIV in Madrid. Pediatr Infect Dis J. 2022; 41(10):824–826. 10.1097/INF.0000000000003624 35796220 PMC9508938

[8] Nosek CA, Buck WC, Caviness AC, et al. Hospital admissions from a pediatric HIV care and treatment program in Malawi. BMC Pediatr. 2016;16:22. 10.1186/s12887-016-0556-3 26830336 PMC4736238

[9] Getahun MB, Teshome GS, Fenta FA, Bizuneh AD, Mulu GB, Kebede MA. Determinants of severe acute malnutrition among HIV-positive children receiving HAART in public health institutions of North Wollo Zone, Northeastern Ethiopia: Unmatched case-control study. Pediatr Health Med Ther. 2020;11:313–321. 10.2147/PHMT.S267892 PMC7490065 32982539

[10] Moyo S, Ismail F, Van der Walt M, et al. Prevalence of bacteriologically confirmed pulmonary tuberculosis in South Africa, 2017–19: A multistage, cluster-based, cross-sectional survey. Lancet Infect Dis. 2022;22(8):1172–1180. 10.1016/S1473-3099(22)00149-9 35594897 PMC9300471

[11] National Department of Health. 2019 ART clinical guidelines for the management of HIV in adults, pregnancy, adolescents, children, infants and neonates. South Africa: Department of Health; 2020.

[12] World Health Organization. Interim WHO clinical staging of HVI/AIDS and HIV/AIDS case definitions for surveillance: African Region [homepage on the Internet]. World Health Organization; 2005 [cited 2024 Sep 19]. Available from: https://apps.who.int/iris/handle/10665/69058

[13] Van der Zalm MM, Lishman J, Verhagen LM, et al. Clinical experience with severe acute respiratory syndrome coronavirus 2-related illness in children: Hospital experience in Cape Town, South Africa. Clin Infect Dis. 2021;72(12):e938–e944. 10.1093/cid/ciaa1666. Erratum in: Clin Infect Dis. 2022;75(1):182. 10.1093/cid/ciaa1666 33170927 PMC7717210

[14] Anyalechi GE, Bain R, Kindra G, et al. Tuberculosis prevalence, incidence and prevention in a South African cohort of children living with HIV. J Trop Pediatr. 2022;68(6):fmac084. 10.1093/tropej/fmac084 36269203 PMC9986743

[15] Vonasek BJ, Rabie H, Hesseling AC, Garcia-Prats AJ. Tuberculosis in children living with HIV: Ongoing progress and challenges. J Pediatric Infect Dis Soc. 2022;11(Supplement_3):S72–S78. 10.1093/jpids/piac060 36314545

[16] Hesseling AC, Cotton MF, Jennings T, et al. High incidence of tuberculosis among HIV-infected infants: Evidence from a South African population-based study highlights the need for improved tuberculosis control strategies. Clin Infect Dis. 2009;48(1):108–114. 10.1086/595012 19049436

[17] Frigati LJ, Brown K, Cotton MF, Myer L, Zar HJ. Hospitalization in South African adolescents with perinatally acquired HIV on antiretroviral therapy. Pediatr Infect Dis J. 2020;39(11):1035–1039. 10.1097/INF.0000000000002826 33006875 PMC7577961

[18] Vonasek B, Ness T, Takwoingi Y, et al. Screening tests for active pulmonary tuberculosis in children. Cochrane Database Syst Rev. 2021;6(6):CD013693. 10.1002/14651858.CD013693.pub2 34180536 PMC8237391

